# Associations between triarchic traits and mental health symptoms: the role of coping styles as mediators

**DOI:** 10.47626/2237-6089-2023-0625

**Published:** 2025-02-27

**Authors:** Lucas de Francisco Carvalho, Cibelle de Oliveira, Maria Clara Romão Pontes Rolim Garcia, Gisele Magarotto Machado

**Affiliations:** Universidade São Francisco Campinas SP Brazil Universidade São Francisco, Campinas, SP, Brazil.

**Keywords:** Behavioral symptoms, antisocial behaviors, coping behavior, mediating factor

## Abstract

**Objectives:**

We investigated relationships between the triarchic model of psychopathy, coping styles, and externalizing and internalizing symptoms, and verified the mediating effect of coping styles.

**Methods:**

Participants were 957 adults who answered the Triarchic Psychopathy Measure (TriPM), the Inventory of Depression and Anxiety Symptoms Expanded Version (IDAS-II), and the Crime and Analogous Behavior Scale (CAB).

**Results:**

Data were analyzed using four path analyses to test our hypotheses, indicating each triarchic trait is differently associated with psychological symptoms and coping styles. We also observed preferences for some coping styles affecting the association between triarchic traits and psychological symptoms.

**Conclusion:**

Our findings suggest that coping styles only affect the interaction between boldness and distress, as well as between boldness and fear, indicating that specific coping strategies can account for variations in distress and fear linked to boldness.

## Introduction

### Psychopathy and mental health symptoms

Psychopathy is a personality disorder characterized by superficial charm, lack of remorse, low empathy, manipulation, and tendencies toward antisocial behavior.^1^ Although considered a personality disorder, psychopathy is not an explicit diagnosis in essential diagnostic manuals such as the Diagnostic and Statistical Manual of Mental Disorders, 5th edition (DSM-5),^2^ or the International Statistical Classification of Diseases and Related Health Problems, 11th revision (ICD-11).^3^ However, these manuals do include the antisocial/dissocial diagnosis, which was initially created to incorporate features of psychopathy.^4^ The focus on behavioral criteria moved the psychopathy diagnosis away from the antisocial personality disorder diagnosis, although they still have considerable overlap.^5^ Advances in the field have been incorporating features of psychopathy into these diagnostic manuals (e.g., the psychopathy specifier in the DSM-5 and the understanding of personality disorders from a trait-based perspective in the ICD-11),^6,7^ but other models still offer more accurate representations of psychopathy. Several models have been proposed to conceptualize psychopathy,^8-10^ with no consensus on which is the most appropriate. Nevertheless, the triarchic model of psychopathy^10^ has been used as a basis for understanding and studying psychopathy, with the advantages of reconciling different historical perspectives on this disorder, encompassing associated neurobiological aspects associated with it, and facilitating associations with the normal-range of personality.^10-12^

The triarchic model understands psychopathy within three phenotypic domains: disinhibition, meanness, and boldness.^10,12^ The disinhibition domain covers impulsive tendencies, difficulties with planning and control, irresponsibility, and antisocial behaviors. Meanness incorporates characteristics related to interpersonal and affective deficits, such as manipulation, low empathy, and exploitation of others. Boldness refers to low fear, low anxiety, dominance, social efficiency, and adventure-seeking tendencies. Although these domains are related and are characteristics of the same disorder, previous studies indicate that they have different (and even inverse) relationships with external variables, such as internalizing and externalizing symptoms.^13-15^

The disinhibition domain is the most related to psychological symptoms, while boldness can be understood as a protective factor for mental disorders. Disinhibition is associated with higher levels of internalizing symptoms such as anxiety, depression, and suicidal tendencies and externalizing symptoms such as substance use and antisocial behaviors.^13,15^ In contrast, boldness is inversely associated with internalizing symptoms (e.g., anxiety, depressive symptoms, and especially symptoms of phobic disorders^11,14,16^), whereas meanness tends to have positive associations with externalizing symptoms (e.g., antisocial behaviors^14^).

### Psychopathy, symptoms, and coping strategies

Previous empirical findings provide a robust basis for the associations between the domains of psychopathy and externalizing and internalizing symptoms. Several individual characteristics, including coping strategies, can impact these relationships. Although the associations between coping and personality traits have already been investigated, including normal-range studies^17,18^ and pathological traits,^19-22^ research focusing on the relationship between coping and typical traits of specific personality disorders, such as psychopathy, is still lacking.

Coping strategies refer to ways of dealing with stressful situations, which can be divided into three main styles^23^: task-oriented, which focuses on solving the problem or changing the situation; emotion-oriented, which represents a tendency to deal with the situation based on aroused emotions; and avoidance-oriented, which reflects the tendency to avoid the stressful situation. The avoidance-oriented style can be separated into two subtypes: social diversion, which refers to shifting the problem focus to socializing with others, and distraction, a tendency to replace the problem focus with an emphasis on other activities.

Specific relationships between coping and psychopathy have been the subject of only a tiny group of empirical studies. Nowakowski and Wróbel^24^ found that boldness is positively associated with greater task-oriented coping style use, while disinhibition is more related to emotion-oriented coping. Saltoğlu and Uysal Irak^25^ investigated the mediating role of coping styles in the relationship between psychopathy and well-being (depression, anxiety, stress, and life satisfaction). In this study,^25^ they investigated psychopathy based on a division between primary and secondary psychopathy.^9^ These authors found that people with high levels of secondary psychopathy traits tend to use more maladaptive coping styles than those with primary psychopathy traits. Furthermore, they also found partial mediating effects of task-oriented, emotion-oriented, and avoidance-oriented styles on the relationships between primary psychopathy and life satisfaction and stress and between secondary psychopathy and the same outcomes and a total mediating effect on the relationship between primary psychopathy and depression symptoms.

Conceiving the coping strategies as mediators of the relationship between psychopathy traits and internalizing and externalizing symptoms is reasonable, as previous studies indicate direct relationships between psychopathy and coping^24,25^ and between psychopathy and internalizing and externalizing symptoms.^13-15^ Empirical findings indicate direct relationships between coping, internalizing, and externalizing symptoms. The emotion-oriented and avoidance-oriented styles are related to higher levels of internalizing and externalizing symptoms,^26,27^ while the task-oriented style is associated with good psychological adjustment.^28^ However, evidence also suggests that psychological adjustment is more related to being fluid across coping styles than always using the same coping strategy.^29,30^

### Current study

The studies by Nowakowski and Wróbel^24^ and Saltoğlu and Uysal Irak^25^ provided relevant information about the relationship between psychopathy and coping strategies. However, these studies were based on covariations without simultaneous control for the influence of all traits (i.e., traits were independently analyzed),^24^ or did not use the triarchic model as a basis for investigation.^25^ This research aimed to investigate relationships between the domains of the triarchic model of psychopathy, coping styles, and externalizing and internalizing symptoms, in addition to verifying the mediating effect of coping styles on the relationship between triarchic traits and mental health.

We hypothesized that boldness would present negative associations with internalizing symptoms (H1a), meanness would show positive associations with antisocial behavior (H1b), and disinhibition would contribute to positive associations with both internalizing and externalizing symptoms (H1c). Moreover, we expected to observe negative associations between boldness and maladaptative coping styles (i.e., emotion-oriented, social diversion, and distraction) and positive associations with adaptative coping styles (i.e., task-oriented; H2a). Contrasting these hypotheses, we also expected disinhibition to show negative associations with task-oriented and positive associations with maladaptative coping styles (H2b). We also anticipated that task-oriented, social diversion, and distraction styles would mediate the relation between boldness and disinhibition and externalizing symptoms (H3a), while the emotion-oriented coping strategy would mediate the relation between triarchic traits and internalizing symptoms (H3b).

## Material and methods

### Participants and procedure

The full sample for the study consisted of 957 Brazilian adults recruited via social media. A Google Forms link for the study survey was shared on Facebook and WhatsApp, inviting individuals to participate and relying on the snowball principle^31^ to reach a large number of participants. The online survey conformed to the standards for conducting and reporting web-based surveys recommended by the Checklist for Reporting Results of Internet E-Surveys (CHERRIES).^32^ All procedures complied with Declaration of Helsinki provisions regarding research with human participants^33^ and were approved by the ethics committee at Universidade São Francisco (CAAE 48338721.9.0000.5514). All participants provided written informed consent before participating.

The full sample (n = 957) was composed mainly of women (87%), and the mean age was 30.3 years (standard deviation [SD] = 11.2, range = 18 to 76). We employed a robust variation of the Mahalanobis distance exclusion method based on the minimum covariance determinant (MCD), the Mahalanobis-MCD,^34^ to enhance the data quality. We used the MCD50 (i.e., a sub-sample of h = n/2 and a breakdown point of 0.5). This method identified 95 multivariate outliers. The final dataset comprised 862 adults ranging from 18 to 76 years (M = 30.6; SD = 11.4) as a result of the exclusions. The majority of the sample reported being women (87.5%), Caucasian (68.8%), single (54.8%), and having spent 13 to 16 years in education (34.3%). Table 1 presents the demographic data for the final sample.

**Table 1:** Final sample descriptive statistics

Variables	n	%
Sex		
Female	754	87.5
Male	108	12.5
		
Years of education		
Fewer than 9	4	0.5
9	7	0.8
From 9 to 11	30	3.5
12	127	14.7
From 13 to 16	296	34.3
17	168	19.5
From 18 to 19	50	5.8
More than 19	180	20.9
		
Ethnicity/skin color		
Caucasian	593	68.8
Brown	194	22.5
Black	52	6.0
Asian	19	2.2
Indigenous	4	0.5
		
Marital status		
Single	472	54.8
Married	303	35.2
Divorced	49	5.7
Widowed	33	3.8
Others	5	0.6

### Measures

#### Triarchic Psychopathy Measure (TriPM)^35^

The TriPM is a 58-item self-report measure for assessing the three traits described by the triarchic model of psychopathy: boldness, meanness, and disinhibition. Items are answered on a four-point Likert scale, from 0 = false to 3 = true. Previous findings support the psychometric properties and convergent and discriminant validity of the TriPM.^36^ We administered a Brazilian Portuguese translation of the TriPM.^37,38^ The reliability of the subscales in this sample was good: Cronbach’s α varied from 0.81 to 0.90 and McDonald’s ω from 0.81 to 0.89.

#### Inventory of Depression and Anxiety Symptoms Expanded Version (IDAS-II)^39^

The IDAS-II is a 99-item self-report measure used to assess internalizing symptomatology. Items are answered on a five-point Likert scale, ranging from 1 = not at all to 5 = extremely. The IDAS-II includes 18 content-based subscales, for which exploratory factor analyses have revealed a three-factor latent structure: distress, obsessions/fear, and positive mood.^39^ Our study used a Brazilian version of the IDAS-II,^40^ and the factors presented good reliability: Cronbach’s α varied from 0.70 to 0.93 and McDonald’s ω from 0.70 to 0.93. Previous studies support the psychometric properties of this measure.^41^

#### Crime and Analogous Behavior Scale (CAB)^42^

The CAB is a 55-item self-report inventory used to assess lifetime externalizing problems related to antisocial behavior and substance use, answered using a two-point response format (0 = no, 1 = yes). To reduce participant fatigue, we administered an abbreviated 16-item version used in prior research^43^ that includes items from the CAB’s Substance Abuse (α = 0.64; ω = 0.66) and Antisocial Behavior subscales (α = 0.63; ω = 0.56). We used a Brazilian version of the CAB scale.^40^

#### The Coping Inventory of Stressful Situations (CISS)^23^

The CISS is a self-report measure to assess the extent to which respondents adhere to different coping styles during stressful situations. This inventory comprises 48 items that are responded on a five-point Likert scale, ranging from 1 = never to 5 = extremely. We translated the CISS into Brazilian Portuguese. Using exploratory structural equation modeling (ESEM) on data from the current sample, we found a four-factor structure with acceptable fit indices. The factors were emotion, task, distraction, and social diversion. Cronbach’s α for items on the subscales composing the four factors in the current study ranged from 0.74 to 0.91, with McDonald’s ω from 0.75 to 0.91. The translation procedures used for this inventory and the results of the internal structural analysis of data from the current sample are detailed in the Supplementary Material S1. Supplementary Table S1 presents the factor structure and factor loadings of the Brazilian Portuguese version of the CISS.

## Data analysis

We calculated descriptive statistics for all variables used in the study, including an investigation of the normality of data using skewness and kurtosis statistics as criteria (i.e., values from -2 to +2 indicate normal distribution^44^). We performed four path analyses to test our hypotheses: a) triarchic traits predict internalizing and externalizing symptoms; b) triarchic traits predict coping styles; c) coping styles predict internalizing and externalizing symptoms; and d) coping styles mediate the association between triarchic traits and internalizing and externalizing symptoms. The path analyses were performed in Mplus Version 7^45^ using the maximum likelihood with robust standard errors (MLR) estimator. We used the following fit indices: the comparative fit index (CFI) and the Tucker-Lewis index (TLI) (≥ 0.95 indicates a good fit, and ≥ 0.90 indicates an acceptable fit) and the root mean square error of approximation (RMSEA) (values < 0.06 are considered good and < 0.10 are considered acceptable).^46-48^ We adopted p < 0.05 as the significance level in this study. The present study was not pre-registered. All data and codes are publicly available from the OSF repository and can be accessed at https://osf.io/ksmxn/?view_only=41e86338e6f84022b56 2a06ce750ed48.

## Results

Table 2 presents the descriptive statistics for the variables used in the study. Most variables tended to be normally distributed.

**Table 2:** Descriptive statistics of the study variables

	Min	Max	Mean	SD	Skewness	Kurtosis
Boldness (TriPM)	1.00	49.00	24.39	9.03	0.01	-0.42
Meanness (TriPM)	0.00	32.00	10.83	7.50	0.73	-0.24
Disinhibition (TriPM)	2.00	49.00	23.23	9.22	0.38	-0.41
Task-oriented (CISS)	1.00	5.00	3.24	0.81	-0.02	-0.46
Emotion (CISS)	1.00	5.00	3.51	0.83	-0.52	-0.44
Distraction (CISS)	1.00	5.00	3.30	0.87	-0.11	-0.44
Social diversion (CISS)	1.00	5.00	2.71	0.86	0.34	-0.27
Substance abuse (CAB)	0.00	6.00	1.51	1.30	0.81	0.23
Antisocial behavior (CAB)	0.00	9.00	0.66	1.01	2.33	9.28
Distress (IDAS-II)	1.00	4.98	2.95	0.94	-0.09	-0.88
Fear (IDAS-II)	1.00	5.00	2.68	0.93	0.20	-0.73
Positive mood (IDAS-II)	1.00	4.67	2.09	0.67	1.05	1.27

CAB = Crime and Analogous Behavior Scale; CISS = Coping Inventory of Stressful Situations; IDAS-II = Inventory of Depression and Anxiety Symptoms Expanded Version; Max = maximum; Min = minimum; SD = standard deviation; TriPM = Triarchic Psychopathy Measure.

Only the antisocial behavior variable deviated from normality. However, the maximum likelihood with robust standard errors (MLR) estimator applied in the path analysis allows non-normally distributed variables.

**Figure 1:**
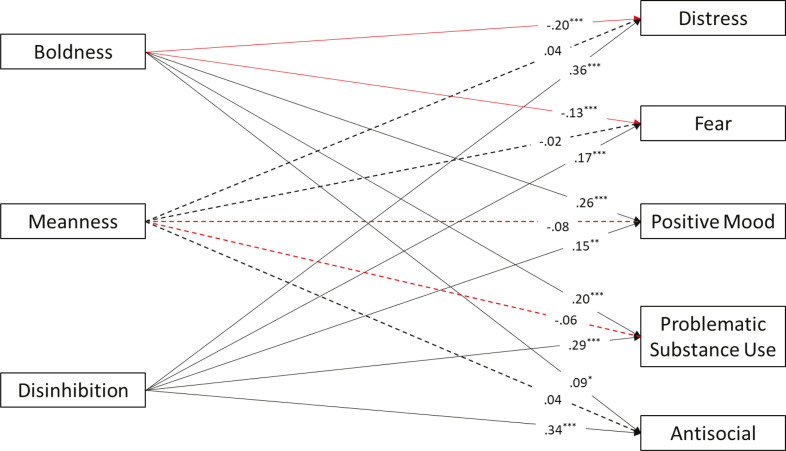
Path model examining triarchic traits as predictors of internalizing and externalizing symptoms. Regression coefficients are standardized βs. For ease of interpretation, dotted lines represent non-significant paths, continuous lines represent significant paths, black lines represent positive associations, and red lines represent negative associations. R2 for dependent variables: distress (R 2= 0.23, p < 0.001); obsessions/fear (R2 = 0.06, p < 0.001); positive mood (R2 = 0.06, p < 0.001); problematic substance use (R2 = 0.07, p < 0.001); antisocial behavior (R2 = 0.12, p < 0.001). *** p < 0.001; ** p < 0.01; * p < 0.05.

We conducted a path analysis to verify the relationship between triarchic traits and internalizing and externalizing psychological symptoms. Figure 1 illustrates the results. The model was just identified (CFI = 1; TLI = 1; RMSEA = 0).

**Figure 2:**
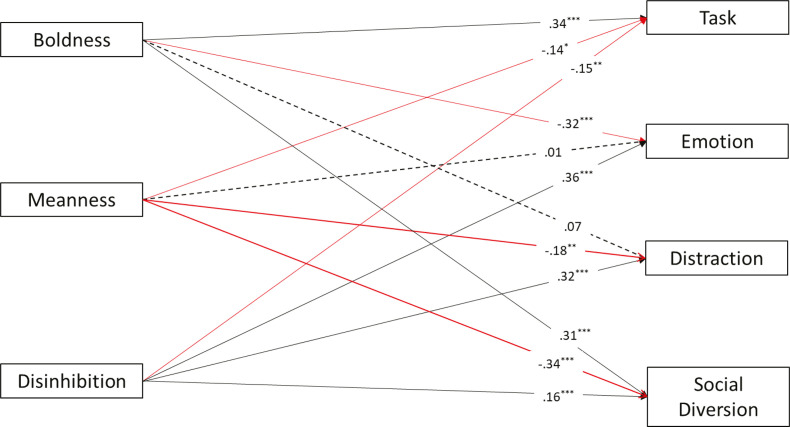
Path model examining triarchic traits as predictors of coping styles. Regression coefficients are standardized βs. For ease of interpretation, dotted lines represent non-significant paths, continuous lines represent significant paths, black lines represent positive associations, and red lines represent negative associations. R2 for dependent variables: task-oriented (R2 = 0.18, p < 0.001); emotion-oriented (R2 = 0.27, p < 0.001); distraction (R2 = 0.05, p < 0.001); problematic substance use (R2 = 0.09, p < 0.001). *** p < 0.001; ** p < 0.01; * p < 0.05.

Meanness was not a significant predictor of any of the symptoms. Boldness was negatively associated with distress and fear, and had positive relations with positive mood, problematic substance use, and antisocial behavior. Disinhibition had positive associations with all internalizing and externalizing symptoms. We conducted a second path analysis to investigate associations between triarchic traits and coping styles. The model was just identified (CFI = 1; TLI = 1; RMSEA = 0). Figure 2 presents the results.

**Figure 3:**
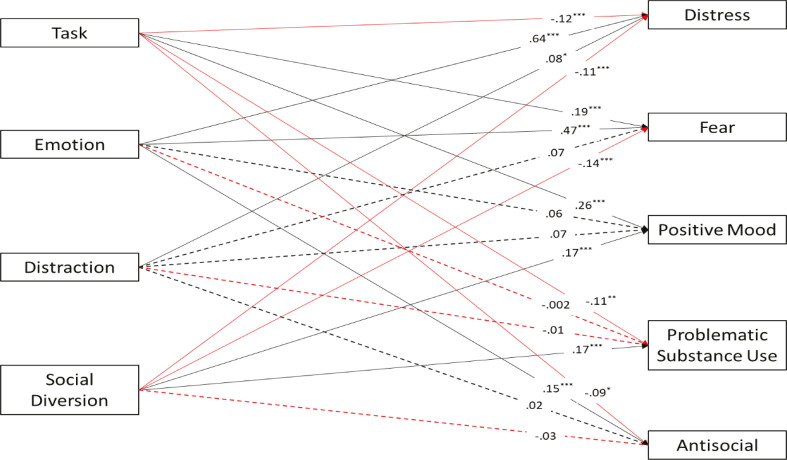
Path model examining coping styles as predictors of psychological symptoms. Regression coefficients are standardized βs. For ease of interpretation, dotted lines represent non-significant paths, continuous lines represent significant paths, black lines represent positive associations, and red lines represent negative associations. R2 for dependent variables: distress (R2 = 0.47, p < 0.001); obsessions/fear (R2 = 0.28, p < 0.001); positive mood (R2 = 0.17, p < 0.001); problematic substance use (R2 = 0.02, p = 0.015); antisocial behavior (R2 = 0.03, p = 0.002). *** p < 0.001; ** p < 0.01; * p < 0.05.

Boldness presented positive associations with task-oriented and social diversion coping styles, negative associations with emotion-oriented coping, and a non-significant association with the distraction style. Meanness was negatively related to all coping styles, except emotion-oriented, for which we observed a non-significant association. Nevertheless, we found a different pattern for disinhibition, which presented positive relations with emotion-oriented, distraction, and social diversion styles, and a negative association with the task-oriented style. We again relied on path analysis to test associations between coping styles and psychological symptoms. Figure 3 shows the results.

The model presented in Figure 3 was just identified (CFI = 1; TLI = 1; RMSEA = 0). We observed significant associations between task-oriented coping and all psychological symptoms in the path model (positive for fear and positive mood, and negative for distress, problematic substance use, and antisocial behavior). Emotion-oriented coping was positively related to all psychological symptoms except positive mood and problematic substance use, for which non-significant associations emerged. Distraction only had a significant association with distress. For social diversion, negative associations emerged with distress and fear, positive associations with positive mood and problematic substance use, and a non-significant association with antisocial behavior.

**Figure 4:**
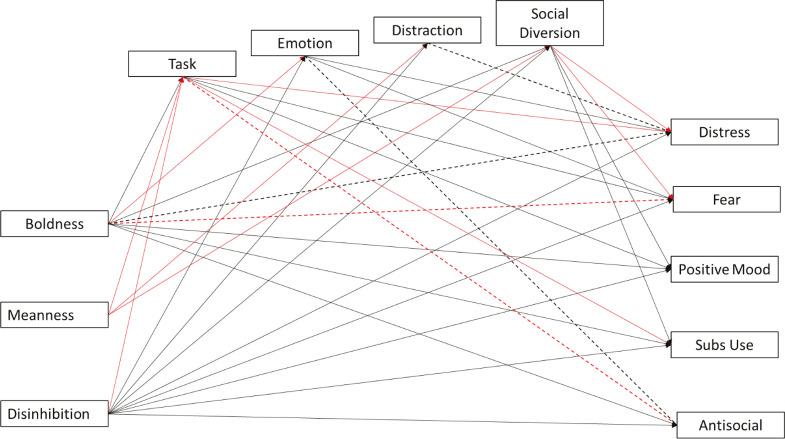
Path model with triarchic traits and coping styles explaining psychological symptoms. Regression coefficients (standardized βs) are not shown in the figure for simplicity. For ease of interpretation, dotted lines represent non-significant paths, continuous lines represent significant paths, black lines represent positive associations, and red lines represent negative associations. R2 for dependent variables: distress (R2 = 0.48, p < 0.001); obsessions/fear (R2 = 0.26, p < 0.001); positive mood (R2 = 0.18, p < 0.001); problematic substance use (R2 = 0.08, p < 0.000); antisocial behavior (R2 = 0.13, p < 0.000). *** p < 0.001; ** p < 0.01; * p < 0.05.

Lastly, we conducted a path model with all triarchic traits, psychological symptoms, and coping styles to verify the effects between triarchic traits and psychological symptoms after accounting for the contribution of coping styles. We excluded paths that had been non-significant in the three previous models before testing this model. Figure 4 presents the results.

The path model presented in Figure 4 has good fit indices (CFI = 0.998; TLI = 0.991; RMSEA = 0.022). The direct relationships between variables were consistent with those observed in the three previous models. Changes were observed in the associations between boldness, distress, and fear and between antisocial, task-oriented, and emotion-oriented coping styles (significant in previously tested models [Figures 1 and 3]). As the direct association between boldness and fear and distress ceased to be significant with the insertion of coping styles in the model, we tested the mediating effect of coping styles within these relations. Significant indirect effects were observed for boldness via task-oriented coping (β = -0.02, p = 0.016), emotion-oriented coping (β = -0.19, p < 0.000), and social diversion coping (β = -0.03, p = 0.002) to predict distress. Significant indirect effects were also observed for boldness via task-oriented coping (β = -0.08, p < 0.000), emotion-oriented coping (β = -0.13, p < 0.000), and social diversion coping (β = -0.03, p = 0.005) to predict fear.

## Discussion

Although the direct relations between psychopathy and psychological symptoms and between psychological symptoms and coping strategies are widely reported in the literature, the associations between psychopathy and coping strategies and the role of coping strategies in the association between psychopathy and mental health still need to be explored. We aimed to investigate the associations between psychopathic triarchic traits, coping styles, and (externalizing and internalizing) symptoms. We also verified the possible mediating effect of coping styles on the relationship between triarchic traits and mental health. Our findings suggest that each triarchic trait is differently associated with psychological symptoms and coping styles. A preference for some coping styles can affect the association between triarchic traits and psychological symptoms.

The findings from the first path model showed boldness positively predicting positive mood and externalizing symptoms (i.e., problematic substance use, antisocial behavior) and negatively predicting internalizing symptoms (i.e., distress and fear); meanness did not predict any of the symptoms; and disinhibition positively predicted all the internalizing and externalizing symptoms, partially confirming our H1. As we predicted in H1a, boldness showed negative associations with internalizing symptoms, indicating a protective role against psychopathologies characterized by anxiety, depression, and suicidality symptoms, as widely reported in the literature.^11,14,16^ However, we did not expect the positive associations between boldness and externalizing symptoms observed in our empirical model. These findings may reflect the mixed results reported previously.^13,49-53^ We also expected positive associations between meanness and antisocial behavior (H1b), which were not confirmed. Although the majority of previous studies had reported these positive associations,^13,53,54^ a recent study conducted in Brazil found non-significant associations between meanness and externalizing symptoms,^40^ which could have been due to the prevalence of women in the sample, similar to our study. Regarding our H1c, we observed all the expected associations between disinhibition and psychological symptoms, supporting the notion that disinhibition is a risk factor for several different forms of psychopathology.^10,11,14^

In our second path model, we expected (H2a) to find negative associations between boldness and maladaptative coping styles (i.e., emotion-oriented, social diversion, and distraction styles) and positive associations with adaptative coping styles (i.e., task-oriented coping). This hypothesis was partially supported, as we found positive associations between boldness and task-oriented coping and a negative association with emotion-oriented coping. However, we also observed an unexpected positive association with the social diversion style. A positive association between boldness and the task-oriented coping style was previously observed,^24^ identifying boldness as the most adaptive of the triarchic traits.^10,55^ Associations with task-oriented style suggest a more adaptive nature for boldness, as this coping style refers to a healthy way of dealing with stressful situations, i.e., it focuses on solving the problem or changing the situation.^23^ The negative association with emotion-oriented coping can also be interpreted in the same light, as this is a maladaptive coping strategy. Although we did not expect the positive association between boldness and the social diversion style (i.e., shifting the problem focus to socializing with others), due to its maladaptative inclination, this finding can be understood given the social potency feature of boldness, which refers to social influence, ability to manipulate and convince others,^10^ and high levels of extraversion.^56^

Moreover, in the second path model, we predicted that disinhibition would be negatively associated with task-oriented and positively associated with maladaptative coping styles (H2b). Our findings support this hypothesis, since disinhibition demonstrated all of the expected associations with the coping strategies. Nowakowski and Wróbel^24^ observed that disinhibition presented the strongest associations with emotion-oriented coping, which was also the strongest association (β = 0.36) observed in the present study. Saltoğlu and Uysal Irak^25^ reported that people with high levels of secondary psychopathy traits (closely related to disinhibition^55^) tend to use maladaptative coping styles, as observed in our results. This finding is compatible with the assumption that disinhibition is the triarchic trait most related to negative outcomes, stressing its maladaptative disposition.^10,11,14^ Although not hypothesized, we observed a milder negative association between meanness and task-oriented and social diversion styles and a strong negative association with distraction, indicating that people with high levels of meanness are less prone to replacing the problem focus by emphasizing other activities.^23^ Furthermore, we can understand that people with low meanness tend to focus on solutions, socializing with people, and different activities as strategies to deal with problems.

Our third path model demonstrated specific associations for each coping style. The task-oriented coping style showed negative associations with most negative outcomes, emerging with a positive association with fear/obsessions only. We may understand these associations in terms of the adaptive nature of the task-oriented style and the ordering, checking, and cleaning features of the fear/obsession factor,^39^ which are related to task execution. The emotion-oriented style was confirmed to be the coping style most associated with psychological symptoms, predicting higher levels of distress, fear/obsessions, and antisocial behavior. Previous studies identified the emotion-oriented style as the most maladaptive coping style.^23,57^ This style is often presented by people with high levels of internalizing symptoms.^27^ Distraction only had a positive association with distress, while social diversion had positive associations with both positive mood and problematic substance use. Silva et al.^58^ suggest that the avoidance-oriented style is more related to externalizing psychopathology, as represented by the problematic substance use associated with the social diversion style. However, the distraction style tends to internalize psychopathology, specifically distress, indicating that people who tend to deal with stressful situations by emphasizing other activities^23^ also present higher levels of depression, anxiety, and suicidality symptoms.^26,27^

To our knowledge, our fourth model constitutes an unprecedented attempt to test the mediating effect of coping styles on the relationship between psychopathic traits and externalizing and internalizing symptoms. Specifically, we expected task-oriented, social diversion, and distraction coping styles to exert mediating effects in the relationship between boldness and disinhibition and externalizing symptoms (H3a); and emotion-oriented coping to exert a mediating effect in the relationship between triarchic traits and internalizing symptoms (H3b). However, coping styles did not impact most associations between traits and symptoms. The only relationships affected were those between boldness and distress and boldness and fear, indicating that these associations can change when we account for the effect of coping styles. These findings suggest that people with high boldness may present less distress due to the tendency to rely more on the task-oriented and social diversion coping styles and to employ emotion-oriented coping less. Similarly, our results imply that people with high boldness could present higher levels of fear if engaged in task-oriented coping, or lower levels of fear due to a tendency to use a social diversion strategy and not to use the emotion-oriented style.

Notwithstanding this study’s contributions regarding the associations of triarchic traits with coping styles and regarding the impact of coping styles on the relationships between psychopathy traits and mental health symptoms, our findings must be pondered in the light of its limitations. First, our sample was recruited online and may not represent the Brazilian population. Besides, we must also consider differences between our sample and samples from previous studies when comparing findings. Our sample was predominantly characterized by adult females from the general population, while prior studies were conducted with adolescents,^59^ incarcerated individuals (e.g.), etc.^52,60^ Second, since our data were based only on participants’ self-report, we did not have information on our sample’s internalizing, externalizing, or psychopathy diagnoses. Third, our study was performed with cross-sectional data, which does not allow directional inferences. We suggest future studies be carried out using truly clinical samples and a longitudinal design strategy.

Despite this study’s limitations, we have contributed additional findings regarding the associations between triarchic traits, mental health symptoms, and coping styles. We believe that one of this study’s strengths is that it is the first, to our knowledge, to test a model containing triarchic traits predicting coping styles simultaneously, therefore controlling the variance of all the predictors in the model. Besides, we are also the first to investigate the mediating effects of coping styles in the relationship between triarchic traits and mental health. Our findings suggest that coping styles only affect the association between boldness and distress and fear, indicating that specific coping strategies can account for the elevation or attenuation of distress and fear linked to boldness.
