# Supplementary material for: Associations between triarchic traits and mental health symptoms: the role of coping styles as mediators
Source: Trends Psychiatry Psychother. 2025 Feb 27;47:e20230625. doi: 10.47626/2237-6089-2023-0625 (PMC12611331; doi:10.47626/2237-6089-2023-0625)
Supplement: Supplementary file 1 [file 2238-0019-trends-47-e20230625_suppl01.pdf]

http://doi.org/10.47626/2237-6089-2023-0625

Supplementary Material S1

We translated the Coping Inventory of Stressful Situations (CISS, Endler and Parker[23]) to Brazilian Portuguese for the purposes of this study. We are presenting the procedures used to translate the scale and investigate its internal structure.

Translation procedures

Initially, two Brazilian authors of this study translated the items independently from the original English to Brazilian Portuguese. We combined the two versions to create a single version of the translated scale. A third Brazilian author performed a back-translation of the items from Brazilian Portuguese to English. The back-translated CISS items were sent to a fourth Brazilian author to evaluate the adequacy of back-translated items against the original items. We reformulated items identified as inappropriate. This process was repeated until further item refinement was deemed unnecessary.

Structural analysis

Initially, we performed a confirmatory factor analysis with the weighted least squares mean and variance adjusted (WLSMV) estimator, replicating the factor structure found by Endler and Parker.[23] The results indicated poor fit indices (CFI = 0.78; TLI = 0.77; RMSEA = 0.082). Given the inadequacy of this structure, we used parallel analysis and ESEM to find the best internal structure for the Brazilian-Portuguese version of the CISS. Parallel analysis indicated the existence of up to five factors. We performed ESEM with one to five factors, applying a WLSMV estimator and Geomin rotation. The four-factor model presented the best interpretability and adequate fit indices: CFI = 0.92, TLI = 0.90, and RMSEA = 0.05. However, this four-factor solution included three items with factor loadings lower than 0.30 on all factors. We excluded these items (3, 11, and 44) and ran a new four-factor solution. The four-factor structure without the three items also showed good fit indices and was retained for further analysis. Supplementary Table S1 shows the factor structure and factor loadings of the Brazilian Portuguese version of the CISS.

http://doi.org/10.47626/2237-6089-2023-0625

Supplementary Table S1 - Factor structure and factor loadings of the Coping Inventory of Stressful Situations (CISS), Brazilian Portuguese version

	Emotion	Task	Distraction	Social diversion
CISS5	**0.71**	-0.01	-0.15	0.10
CISS7	**0.46**	0.22	0.08	0.08
CISS8	**0.81**	-0.03	-0.09	-0.02
CISS13	**0.86**	0.00	0.08	-0.15
CISS14	**0.81**	0.02	0.13	-0.23
CISS16	**0.49**	-0.01	0.17	-0.01
CISS17	**0.78**	-0.03	0.05	-0.03
CISS19	**0.74**	-0.05	0.23	-0.18
CISS22	**0.85**	-0.10	-0.05	0.00
CISS25	**0.59**	-0.29	0.01	0.07
CISS28	**0.77**	0.02	-0.01	-0.03
CISS30	**0.68**	0.26	-0.06	0.14
CISS33	**0.47**	0.14	0.03	0.11
CISS34	**0.59**	0.23	-0.10	0.12
CISS38	**0.56**	0.00	0.33	-0.18
CISS45	**0.40**	-0.13	0.30	-0.09
CISS1	-0.23	**0.68**	0.00	-0.04
CISS2	-0.08	**0.74**	-0.03	-0.06
CISS6	0.08	**0.44**	0.14	-0.01
CISS10	-0.05	**0.70**	0.12	-0.08
CISS15	0.25	**0.57**	0.09	-0.03
CISS21	-0.10	**0.73**	0.11	-0.03
CISS24	0.07	**0.68**	-0.07	0.11
CISS26	-0.15	**0.66**	0.10	-0.02
CISS27	0.01	**0.69**	-0.07	0.10
CISS36	-0.05	**0.73**	-0.15	0.09
CISS39	0.00	**0.79**	-0.01	0.04
CISS41	-0.09	**0.78**	0.01	0.02
CISS42	0.20	**0.63**	0.02	0.05
CISS43	0.13	**0.80**	-0.05	0.00
CISS46	0.10	**0.50**	0.16	0.05
CISS47	0.02	**0.60**	0.16	-0.07
CISS9	-0.05	0.08	**0.66**	0.04
CISS12	0.06	0.11	**0.65**	0.11
CISS18	-0.01	-0.01	**0.59**	0.32
CISS20	0.04	0.00	**0.81**	0.09
CISS40	0.18	0.19	**0.32**	0.12
CISS48	0.09	0.08	**0.32**	0.08
CISS4	-0.04	0.12	0.06	**0.57**
CISS23	-0.11	-0.14	0.29	**0.62**
CISS29	0.00	-0.06	0.10	**0.83**
CISS31	0.08	0.10	0.19	**0.48**
CISS32	-0.09	0.23	0.02	**0.42**
CISS35	0.16	0.24	-0.03	**0.56**
CISS37	0.02	0.14	0.00	**0.67**

Loadings ≥ 0.30 are in bold.
